# Imaging and Impact of Myocardial Fibrosis in Aortic Stenosis

**DOI:** 10.1016/j.jcmg.2018.11.026

**Published:** 2019-02

**Authors:** Rong Bing, João L. Cavalcante, Russell J. Everett, Marie-Annick Clavel, David E. Newby, Marc R. Dweck

**Affiliations:** a British Heart Foundation Centre for Cardiovascular Science, University of Edinburgh, Edinburgh, United Kingdom; b Division of Cardiovascular Diseases, Department of Medicine, UPMC Heart & Vascular Institute, University of Pittsburgh, Pittsburgh, Pennsylvania; c Quebec Heart & Lung Institute, Laval University, Quebec City, Quebec, Canada

**Keywords:** aortic stenosis, cardiac magnetic resonance, late gadolinium enhancement, myocardial fibrosis, T_1_ mapping, AVR, aortic valve replacement, CI, confidence interval, CMR, cardiac magnetic resonance, CT, computed tomography, ECV%, extracellular volume fraction, HR, hazard ratio, iECV, indexed extracellular volume, LGE, late gadolinium enhancement, SAVR, surgical aortic valve replacement, TAVR, transcatheter aortic valve replacement

## Abstract

Aortic stenosis is characterized both by progressive valve narrowing and the left ventricular remodeling response that ensues. The only effective treatment is aortic valve replacement, which is usually recommended in patients with severe stenosis and evidence of left ventricular decompensation. At present, left ventricular decompensation is most frequently identified by the development of typical symptoms or a marked reduction in left ventricular ejection fraction <50%. However, there is growing interest in using the assessment of myocardial fibrosis as an earlier and more objective marker of left ventricular decompensation, particularly in asymptomatic patients, where guidelines currently rely on nonrandomized data and expert consensus. Myocardial fibrosis has major functional consequences, is the key pathological process driving left ventricular decompensation, and can be divided into 2 categories. *Replacement fibrosis* is irreversible and identified using late gadolinium enhancement on cardiac magnetic resonance, while *diffuse fibrosis* occurs earlier, is potentially reversible, and can be quantified with cardiac magnetic resonance T_1_ mapping techniques. There is a substantial body of observational data in this field, but there is now a need for randomized clinical trials of myocardial imaging in aortic stenosis to optimize patient management. This review will discuss the role that myocardial fibrosis plays in aortic stenosis, how it can be imaged, and how these approaches might be used to track myocardial health and improve the timing of aortic valve replacement.

## Central Illustration

**Figure:**
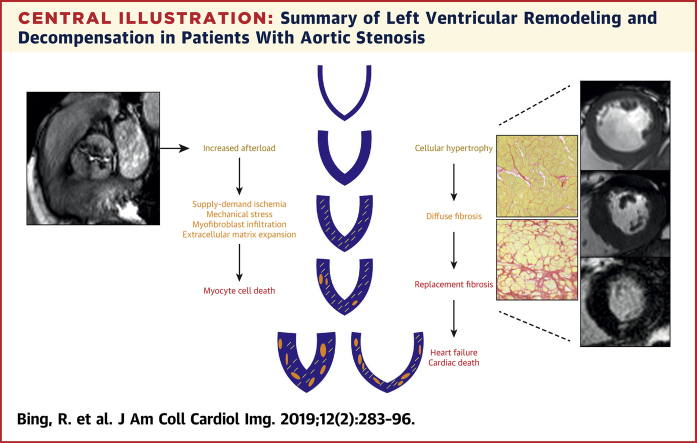

Aortic stenosis is one of the most common valvular diseases in the Western world 1, 2, with an estimated prevalence as high as 12.4% in the elderly (3). Aortic stenosis is characterized not only by progressive valve obstruction, but also by the left ventricular remodeling response (4). Narrowing of the valve causes pressure overload of the left ventricle and triggers a hypertrophic response that maintains myocardial performance for many years, if not decades. However, with time, this process decompensates as patients transition from hypertrophy to heart failure, a change that is heralded clinically by the development of symptoms and adverse events, leading to consideration of aortic valve replacement (AVR).

Aortic stenosis progresses inexorably. Although the early stages are asymptomatic and associated with a good prognosis, advanced disease is associated with substantial morbidity and mortality 5, 6, 7. Despite much research, to date there are no proven medical therapies that slow disease progression. The only definitive treatment for severe aortic stenosis remains AVR, either by surgical aortic valve replacement (SAVR) or transcatheter aortic valve replacement (TAVR) approaches. The uptake of TAVR has grown exponentially 3, 8, as interventions that were initially offered only to elderly, inoperable patients are now being performed in younger, lower-risk patients with excellent results 9, 10, 11, 12, 13. Decisions about if, when, and how to intervene have therefore become increasingly complex, requiring careful assessment of individual patients within a multidisciplinary heart team.

Current guidelines recommend intervention in patients with severe aortic stenosis and evidence of left ventricular decompensation. Most commonly this is in the form of development of typical symptoms, but other markers include a reduction in ejection fraction <50%, an abnormal exercise tolerance test, or a rise in brain natriuretic peptide levels 14, 15. Unfortunately, symptoms are often difficult to identify in the elderly comorbid patients encountered in clinical practice, and many of the other changes appear only late in the course of the disease after irreversible myocardial damage has become established. European Society of Cardiology guidelines provide a Class 1 recommendation, Level of Evidence: B, for intervention in the most common scenario—symptomatic, severe aortic stenosis. However, intervention in asymptomatic patients with a reduction in ejection fraction <50% or an abnormal exercise test is only Level of Evidence: C (i.e., expert opinion) (15). The American College of Cardiology and American Heart Association guidelines are largely in alignment (14). This highlights the need for more robust data to better risk-stratify patients and optimize management strategies before the onset of symptoms and heart failure.

Consequently, there is extensive interest in identifying novel, objective markers of early left ventricular decompensation to optimize the timing of AVR and track myocardial health over time. The development of such markers requires improved understanding of the pathophysiology underling left ventricular decompensation in aortic stenosis. Histological studies have suggested that myocardial fibrosis and cell death are both important drivers of this process 16, 17. Attention has focused on myocardial fibrosis in particular, given its structure-function correlation with heart failure and the fact that it can now be identified reliably and noninvasively with modern imaging techniques. This review will discuss the pathophysiology of myocardial fibrosis and left ventricular decompensation in aortic stenosis, the imaging techniques that can be used to detect it, and how these might be employed to track myocardial health and optimize the timing of AVR.

## Pathology

It is useful to consider aortic stenosis as a disease of both the valve and the myocardium (4). In addition, the importance of arterial stiffness and systemic pulsatile arterial load cannot be underestimated in this elderly population 18, 19. A detailed discussion of events within the valve is beyond the scope of this review (20); however, an understanding of the pathological factors driving the hypertrophic remodeling response and its subsequent decompensation are critical to understanding the rationale for myocardial fibrosis imaging (Central Illustration).

**Central Illustration:**
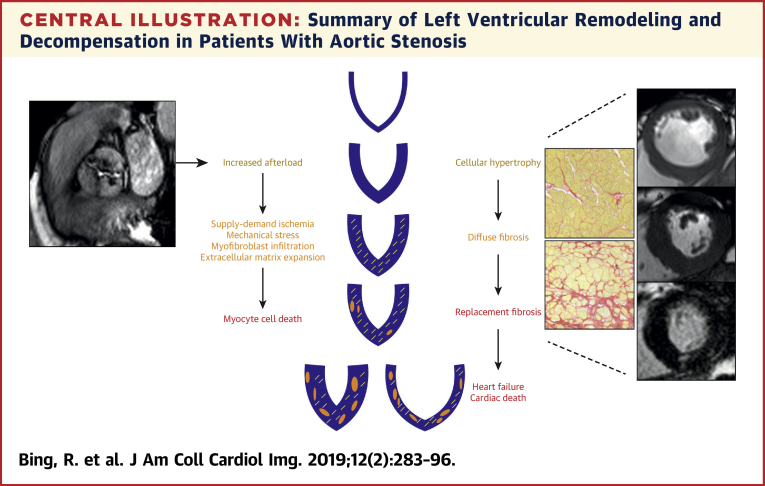
Summary of Left Ventricular Remodeling and Decompensation in Patients With Aortic Stenosis Schematic of the left ventricular remodeling response in aortic stenosis, describing the transition from hypertrophy to fibrosis, heart failure, and cardiac death.

Progressive valve narrowing causes pressure overload of the left ventricle and triggers a hypertrophic response that maintains wall stress and left ventricular performance for many years. Over time, this process decompensates and patients transition from hypertrophy to heart failure, leading to adverse clinical outcomes. This evolution is complex but is closely related to the development of myocardial fibrosis, myocyte injury, and cell death. Furthermore, there is adverse remodeling of the extracellular matrix, with degradation and disruption of the matrix structure (21). These changes are regulated by several factors, including the renin-angiotensin-aldosterone system, transforming growth factor beta, apoptosis signal-regulating kinase 1, and tissue inhibitor of metalloproteinase 22, 23, 24: all potential targets for novel therapeutic interventions.

Two distinct myocardial fibrosis patterns have been described. Reactive interstitial fibrosis is diffuse and follows increased myofibroblast activity and collagen deposition that begins even in the early stages of aortic stenosis. Importantly, this diffuse fibrosis is reversible and has been demonstrated to regress following AVR (16). In contrast, replacement fibrosis appears to occur later and is irreversible (25). Treibel et al. (26) recently demonstrated that patients with advanced disease undergoing AVR manifest a complex combined pattern of both replacement and diffuse fibrosis. Moreover, they observed a fibrosis gradient from the subendocardium to the mid-myocardium, perhaps suggesting supply-demand ischemia as a contributing factor.

The degree of myocardial remodeling and fibrosis is closely related to hemodynamic markers of myocardial performance, such as end-diastolic pressure and ejection fraction (4). Moreover, multiple histological studies have now demonstrated an association between myocardial fibrosis at the time of AVR and both impaired recovery of left ventricular systolic function and poor long-term outcomes following valve replacement 17, 27, 28, 29. Although it is certainly plausible that myocardial fibrosis might directly contribute to such outcomes, a causal relationship is yet to be demonstrated.

## Imaging Modalities for the Assessment of Myocardial Fibrosis

Although myocardial biopsy and histological analysis are still considered the gold standard assessments of myocardial fibrosis, they have several important limitations precluding their routine clinical application. Myocardial biopsy is an invasive procedure that carries an attendant risk of complications (30). Additionally, as only small areas of the myocardium can be sampled, biopsy is prone to sampling error. By contrast, modern imaging techniques, in particular those provided by cardiovascular magnetic resonance (CMR), allow comprehensive, noninvasive assessments of fibrosis across the entire myocardium as well as quantification of its functional consequences (Table 1). These approaches have been used to assess myocardial fibrosis in a range of cardiovascular conditions including aortic stenosis and are described in the following text.

**Table 1:** Performance of Different Imaging Modalities in Aortic Stenosis

	Severity	Ventricular Performance	Diffuse Fibrosis	Replacement Fibrosis	Long-Term Prognosis
TTE	+++	+++	-	-	+++
CT	++	++	+	+	+
CMR	+	+++			
Native T1			++	+	+
ECV%/iECV			++	+	+
LGE			-	+++	+++
FT	-	+++	-	-	+

CMR = cardiac magnetic resonance; CT = computed tomography; ECV% = extracellular volume fraction; FT = feature tracking; iECV = indexed extracellular volume; LGE = late gadolinium enhancement; TTE = transthoracic echocardiogram.

### Cardiac magnetic resonance

CMR provides unparalleled soft tissue characterization and can be used to identify and measure both diffuse and replacement forms of fibrosis in a single scan without the use of ionizing radiation. When utilized together, the CMR techniques described in the following text offer the best available method of capturing the full spectrum of fibrotic changes within the left ventricular myocardium (26).

#### Late gadolinium enhancement

Gadolinium-based contrast agents (GBCAs) partition into areas of extracellular expansion (myocardial edema, necrosis, infiltration, or fibrosis). Interpretation of delayed imaging using GBCAs requires clear differences in signal intensity between healthy and diseased myocardium in a relatively discrete distribution. Consequently, late gadolinium enhancement (LGE) is an excellent marker of focal replacement fibrosis, but is insensitive for the detection of more diffuse interstitial fibrosis.

**Figure 1:**
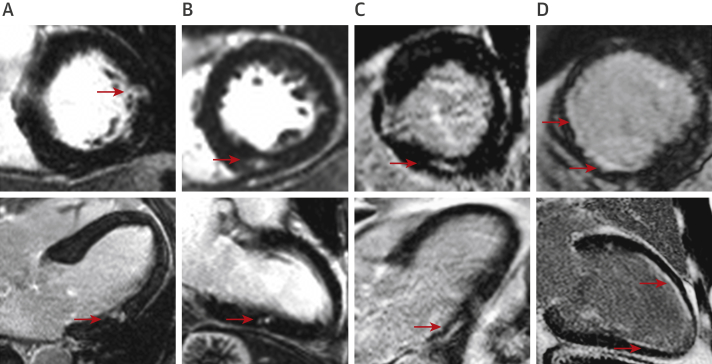
Late Gadolinium Enhancement Patterns in Aortic Stenosis Each panel shows short-axis **(top)** and corresponding long-axis **(bottom)** late gadolinium images from cardiac magnetic resonance scans. **(A to C)** Focal noninfarct late gadolinium enhancement typical of the replacement fibrosis seen in aortic stenosis. **(D)** Subendocardial late gadolinium enhancement in coronary artery territories, consistent with scar due to infarction rather than focal noninfarct fibrosis. Areas of infarction such as these should be excluded when calculating extracellular volume fraction. **Red arrows** indicate areas of late gadolinium enhancement.

LGE is now well established and widely used as a method for detecting replacement myocardial fibrosis in a broad range of cardiovascular conditions such as ischemic cardiomyopathy, nonischemic dilated cardiomyopathy, cardiac sarcoidosis, cardiac amyloidosis, myocarditis, and hypertrophic cardiomyopathy 31, 32, 33, 34, 35, 36, 37, 38. In each condition, replacement fibrosis detected by LGE serves as an independent and powerful predictor of mortality and adverse cardiovascular events. LGE is also the most studied and best validated imaging method for detecting myocardial fibrosis in aortic stenosis. Multiple independent studies have described a noninfarct (or mid-wall) pattern of LGE in patients with aortic stenosis that is distinct from the pattern of scarring seen in other pathologies such as myocardial infarction (Figure 1). On histology, noninfarct LGE co-localizes with microscars and replacement fibrosis, whereas clinical studies have validated it against other markers of left ventricular decompensation and demonstrated a close association with advanced left ventricular hypertrophy, increased myocardial injury, electrocardiographic changes, impaired diastolic and systolic function, and reduced exercise capacity 25, 39, 40, 41. Once noninfarct LGE becomes established, it progresses rapidly. Although the process is arrested by aortic valve intervention, replacement fibrosis appears irreversible once established. Thus, the burden of replacement fibrosis a patient accumulates while waiting for valve intervention persists with them until death (42). The clinical implications are important, as noninfarct LGE is associated with a poor long-term prognosis. Indeed, 5 studies and a recent meta-analysis (43) have confirmed noninfarct LGE to be an independent predictor of mortality, of incremental value to valve assessments, comorbidity, and left ventricular ejection fraction 28, 41, 44, 45, 46 (Table 2).

**Table 2:** CMR Studies Investigating Myocardial Fibrosis in Aortic Stenosis

Study (Ref. #)	Year	n	Population	CMR	Biopsy	Findings
**Native T**_**1**_**Studies**						

Bull et al. (55)	2013	109	Severe AS undergoing SAVR Asymptomatic moderate or severe AS	1.5-T Native T_1_ shMOLLI	19	Native T_1_ correlated with CVF (r = 0.65; p = 0.002) and increased with disease severity.
Lee et al. (56)	2015	80	Asymptomatic moderate or severe AS	3-T Native T_1_ MOLLI	20	Native T_1_ correlated with histology (r = 0.777; p < 0.001) and TTE measures of diastolic dysfunction, and was increased compared with control patients, with overlap.

**ECV Studies**						

Flett et al. (62)	2010	18	Severe AS undergoing SAVR	1.5-T ECV% EQ-CMR FLASH-IR	18	ECV% correlated with CVF (r^2^ = 0.86; p < 0.001).
Fontana et al. (77)	2012	18	Severe AS undergoing SAVR	1.5-T ECV% EQ-CMR shMOLLI FLASH-IR	18	ECV% correlated with CVF (r^2^ = 0.685). ShMOLLI was superior to FLASH-IR.
White et al. (66)	2013	18	Severe AS undergoing SAVR	1.5-T ECV% EQ-CMR DynEQ-CMR shMOLLI	18	ECV% by both methods correlated with CVF (r^2^ = 0.69; p < 0.01 and r^2^ = 0.71; p < 0.01).
Flett et al. (78)	2012	63	Severe AS undergoing SAVR	1.5-T ECV% EQ-CMR FLASH-IR	—	ECV% was increased compared with control subjects, with overlap. At 6 months, LVH had regressed but diffuse fibrosis was unchanged.

**LGE Studies**						

Weidemann et al. (27)	2009	46	Severe AS undergoing AVR	LGE	46	LGE appeared to be concordant with histology (88% with severe fibrosis had ≥2 positive segments; 89% with no fibrosis had no positive segments) and did not regress at 9 months post-AVR.
Azevedo et al. (28)	2010	28	Severe AS undergoing AVR	1.5-T LGE	28	LGE was present in 61%. LGE correlated with histology (r = 0.67; p < 0.001). LGE was an independent predictor of all-cause mortality (HR: 1.26; 95% CI: 1.03–1.54; p = 0.02).
Debl et al. (79)	2006	22	Symptomatic AS	1.5-T LGE	—	LGE was present in 27%. LGE correlated with more severe AS and LVH.
Rudolph et al. (80)	2009	21	Any AS	1.5-T LGE	—	LGE was present in 62%. LGE correlated with increased LV mass and end-diastolic volume index.
Dweck et al. (44)	2011	143	Moderate or severe AS	1.5-T LGE	—	LGE present in 66%. Midwall LGE present in 38%. Midwall LGE was an independent predictor of all-cause mortality (HR: 5.35; 95% CI: 1.16–24.56; p = 0.03).
Baron-Rochette et al. (45)	2014	154	Severe AS undergoing AVR	1.5-T LGE	—	LGE present in 29%. LGE was an independent predictor of all-cause mortality (HR: 2.8; 95% CI: 1.1 to 6.9; p = 0.025).
Rajesh et al. (81)	2017	109	Severe AS	1.5-T LGE	—	LGE present in 43%. Midwall LGE present in 31%. LGE predicted heart failure/hospitalization and a fall in LVEF but did not predict mortality.
Musa et al. (46)	2018	674	Severe AS undergoing AVR	1.5-T, 3-T LGE	—	LGE present in 51%. Noninfarct LGE present in 33%. Scar associated with all-cause (26.4% vs 12.9%; p < 0.001) and cardiovascular (15.0% vs 4.8%; p < 0.001) mortality in a dose-dependent fashion (for every 1% increase in scar, HR: 1.11; 95% CI: 1.05–1.17; p < 0.001 for all-cause and HR: 1.08; 95% CI: 1.01–1.17; p < 0.001 for cardiovascular mortality). Infarct and noninfarct scar were both associated with adverse outcomes.
de Meester et al. (82)	2015	12	Severe AS undergoing SAVR	3-T Native T_1_ ECV% LGE MOLLI	12	LGE was present in 17 of 31 patients (from total cohort). Only ECV% correlated with histology (r = 0.79; p = 0.011).
Kockova et al. (57)	2016	31	Severe AS undergoing SAVR	1.5-T Native T_1_ ECV% MOLLI	31	Patient with severe MF (>30%) on histology had higher native T_1_ times and ECV%. Native T_1_ ≥1,010 ms and ECV ≥0.32 had AUC of 0.82 and 0.85, respectively, for severe MF.
Chin et al. (41)	2017	166	Any AS	3-T iECV LGE MOLLI	11	Midwall LGE was present in 27%. iECV correlated with histology (r = 0.87; p < 0.001) and was increased compared with control subjects. iECV + LGE predicted unadjusted all-cause mortality (36 vs. 8 deaths/1,000; p = 0.009).
Treibel et al. (26)	2018	133	Severe AS undergoing AVR	1.5-T ECV% LGE MOLLI	133	LGE was present in 60%; noninfarct pattern was more common. Complex MF patterns. LGE, but not ECV%, correlated with CVF in all biopsies (r^2 =^ 0.28; p < 0.001) but more in biopsies with endocardium (r^2^ = 0.501; p < 0.001). Combined LGE + ECV% best predicted LV remodeling and functional capacity.
Child et al. (83)	2018	25	Severe AS	3-T Native T_1_ ECV% LGE MOLLI, shMOLLI, SASHA	12	Noninfarct LGE was present in 20%. Sequences differed in discrimination between health and disease as well as association with CVF. Native T_1_ with MOLLI correlated best (r = 0.582; p = 0.027).
Chin et al. (59)	2014	20	Any AS	3-T Native T_1_ ECV% MOLLI	—	ECV displayed excellent scan-rescan reproducibility and was higher in AS than control subjects. Native T_1_ was not as reproducible and was not significantly higher in AS than control subjects.
Chin et al. (40), Shah et al. (39)	2014	122	Any AS	3-T ECV% LGE MOLLI	—	Midwall LGE was present in 28%. ECV% and LGE were associated with elevated TnI and ECG evidence of strain.
Dusenberry et al. (84)	2014	35	Congenital AS	1.5-T ECV% LGE Look-Locker	—	LGE was present in 24%. ECV% was increased compared to control patients and correlated with TTE measures of diastolic dysfunction.
Treibel et al. (25)	2018	116	Severe AS undergoing AVR	1.5-T iECV LGE MOLLI	—	At 1 yr, cellular and matrix volume regressed. LGE was unchanged.
Everett et al. (42)	2018	99	61 asymptomatic AS 38 severe AS undergoing AVR	1.5-T, 3-T iECV LGE	—	Midwall LGE was present in 26%. LGE progressed from baseline and was most rapid in patients with more severe stenosis. In patients undergoing AVR, iECV reduced by 11% (4%–16%) but there was no change in LGE.
Lee et al. (58)	2018	127	Moderate or severe AS	3-T Native T_1_ LGE MOLLI	—	LGE was present in 32.3%. Native T_1_ was increased compared with control patients, with overlap. Native T_1_ and LGE were independent predictors of poor prognosis.

AS = aortic stenosis; AUC = area under the curve; CI = confidence interval; CMR = cardiac magnetic resonance; CVF = collagen volume fraction; DynEQ-CMR = dynamic equilibrium contract-cardiac magnetic resonance; ECV% = extra-cellular volume fraction; EQ-CMR = equilibrium contrast cardiac magnetic resonance; FLASH-IR = fast low angle single shot inversion recovery; HR = hazard ratio; iECV = indexed extracellular volume; LGE = late gadolinium enhancement; LVEF = left ventricular ejection fraction; LVH = left ventricular hypertrophy; MOLLI = modified Look-Locker inversion recovery; SASHA = saturation recovery single-shot acquisition; SAVR = surgical aortic valve replacement; shMOLLI = shortened modified Look-Locker inversion recovery; TnI = troponin I; TTE = transthoracic echocardiography.

The poor prognosis associated with non-infarct LGE appears to persist long after AVR is performed, in keeping with the irreversible nature of replacement fibrosis. In the largest study to date, the British Society for Cardiovascular Magnetic Resonance Valve Consortium performed comprehensive CMR assessments in over 650 patients with severe aortic stenosis just prior to SAVR or TAVR (46). At a median follow-up of 3.6 years, LGE (present in 50% of patients) was a powerful independent predictor of all-cause (26.4% vs. 12.9%; p < 0.001) and cardiovascular mortality (15.0% vs. 4.8%; p < 0.001) following AVR. Furthermore, this association appeared dose-dependent: with every 1% increase in left ventricular myocardial scar burden, all-cause and cardiovascular mortality increased by 11% and 8%, respectively (hazard ratio [HR]: 1.11; 95% confidence interval [CI]: 1.05 to 1.17; p < 0.001; and HR: 1.08; 95% CI: 1.01 to 1.17; p < 0.001). Similar effects were observed for both infarct and noninfarct LGE. Noninfarct LGE was also demonstrated to be an independent predictor of both all-cause and cardiovascular mortality.

LGE is reliable, well-validated, and easily integrated into the standard workflow, with post-processing and qualitative analysis readily performed in <10 min in most cases. LGE is therefore ready for investigation as a tool for use in routine clinical practice. Indeed, the ongoing EVOLVED (Early Valve Replacement Guided by Biomarkers of Left Ventricular Decompensation in Asymptomatic Patients with Severe Aortic Stenosis) trial (NCT03094143) (47) will investigate whether patients in whom noninfarct LGE is identified may benefit from early AVR before further fibrosis develops and left ventricular decompensation progresses (see the Future Directions section).

#### T_1_ mapping

**Figure 2:**
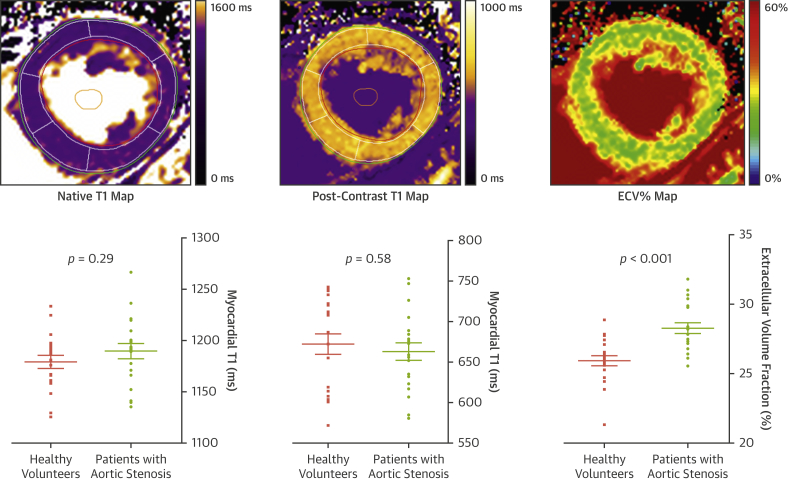
T_1_ Mapping Three different cardiac magnetic resonance T_1_ maps are demonstrated. Native T_1_ and post-contrast T_1_ maps are generated by the signal intensity encoded within each voxel, depending on the T_1_ relaxation time; color coding according to T_1_ times is applied for visual reference. ECV% maps are generated using the formula ECV% = (Δ[1/T1_myo_]/Δ[1/T1_blood_]) × (1 − hematocrit), where Δ(1/T1) is the difference in myocardial or blood T1 pre-contrast and post-contrast. ECV% can be used to assess the proportion of the myocardium comprised by extracellular space. Note that there is significant overlap between health and disease with native and post-contrast T_1_, in contrast to ECV%. Graphs adapted from Chin et al. (59) by permission of Oxford University Press. ECV% = extracellular volume fraction; iECV = indexed extracellular volume.

Although LGE is now well-established as a marker of replacement fibrosis, this technique is not able to detect the diffuse interstitial fibrosis that also characterizes left ventricular decompensation in aortic stenosis. Moreover, LGE quantification can be challenging in diffuse fibrotic states. Novel CMR T_1_ mapping approaches have been developed to overcome these issues. These are reviewed in depth elsewhere 48, 49, but in brief, parametric T_1_ maps are produced where the tissue T_1_ time is encoded as signal intensity within each voxel on a static 2-dimensional image and converted to color maps to aid visual interpretation (Figure 2). Native T_1_ values reflect the state of both the intracellular and extracellular environments, while the addition of a GBCA facilitates targeted interrogation of the extracellular space.

Various protocols for T_1_ mapping have been studied (49). The original Look-Locker technique (50) has been largely superseded by modern variations. The modified Look-Locker imaging sequence (51) is the most studied inversion-recovery technique, whereas variants such as the shortened modified Look-Locker imaging sequence require a shorter breath hold (52). Optimization of protocols has improved accuracy, acquisition time, and ease of use via reduction in heart rate dependence and breath holds. Moreover, post-processing and analysis of T_1_ mapping data can now be performed with fast and reproducible techniques utilizing standardized protocols. T_1_ mapping techniques are now readily accessible in many CMR units and will be discussed below.

#### Native T_1_

As fibrosis increases, native T_1_ values increase. Quantitative T_1_ measurements therefore allow detection of focal or diffuse fibrosis without the use of GBCAs, although the T_1_ signal also changes with other pathological processes such as edema or myocardial infiltration. Native T_1_ has been utilized in conditions such as myocardial infarction, myocarditis, dilated cardiomyopathy, cardiac amyloid, and Fabry disease (48), and has demonstrated significant prognostic power beyond that of LGE alone 53, 54. Although less robust, data is also emerging for native T_1_ in aortic stenosis. Recent studies have demonstrated a correlation between native T_1_ and both the degree of diffuse fibrosis on histology and the extent of ventricular remodeling on CMR 55, 56, 57 (Table 2). Lee et al. (58) recently presented a single-center cohort of 127 patients with moderate or severe AS in whom native T_1_ was an independent predictor of heart failure hospitalization or death (2.4% vs. 11.6% vs. 42.9% for low, mid, and high tertiles of native T_1_, respectively; p < 0.001).

Although native T_1_ is relatively uniform and reproducible when using the same sequence and scanner on the same patient, values are subject to a variety of factors such as patient age and sex, acquisition sequence, scanner field strength, and post-processing. In aortic stenosis, even within the same scanner and protocol, substantial overlap exists in T_1_ values across different severities of aortic stenosis and with healthy control subjects (59). Consequently, there are no universal cutoffs for health and disease in aortic stenosis (60). The International T1 Mapping Multicenter Consortium (61) has successfully standardized a multivendor sequence and provided valuable diagnostic and prognostic data in other disease states. However, although native T_1_ holds major appeal as a marker of diffuse fibrosis that does not require contrast administration and is favored as a technique by various experts in the field, its specific role in aortic stenosis requires further research.

#### Post-contrast T_1_ mapping

GBCAs do not cross cell membranes and therefore distribute throughout the extracellular space in the myocardium. Post-contrast T_1_ mapping techniques therefore allow more specific interrogation of the extracellular space due to gadolinium’s shortening effects on T_1_ relaxation times. Unfortunately, standardization of post-contrast T_1_ mapping values is difficult due to variation in gadolinium kinetics between patients and even within the same individual on different days. Standardized normal values are again lacking, and consequently, post-contrast T_1_ mapping is not in widespread use.

#### Extracellular volume fraction

The extracellular volume fraction (ECV%) corrects post-contrast myocardial T_1_ mapping values for blood pool and pre-contrast myocardial T_1_, thereby accounting for differences in blood concentrations of GBCAs. By incorporating the hematocrit, ECV% calculates the fraction of the myocardium comprised by the extracellular space according to the formula ECV% = (Δ[1/T1_myo_]/Δ[1/T1_blood_]) × (1 − hematocrit), where Δ(1/T1) is the difference in myocardial or blood T_1_ pre- and post-contrast (62). A key feature of myocardial fibrosis is the deposition of excess collagen in the interstitial space and the subsequent expansion of the extracellular space. ECV% has therefore been investigated as a method for detecting diffuse myocardial fibrosis in a range of cardiovascular conditions including myocardial infarction, nonischemic cardiomyopathy, and aortic stenosis 63, 64.

Current scanning techniques assume a dynamic equilibrium between blood and myocardium ∼10 to 15 mins after a bolus injection of contrast 65, 66. A synthetic ECV% has also been described that derives hematocrit from the longitudinal relaxation rate of blood, obviating the need for blood sampling (67), while a more recent noninvasive point-of-care probe to derive hematocrit has demonstrated promising results when compared with both standard and synthetic ECV% (68). ECV% has thus become easier to measure and more clinically applicable. Moreover, ECV% potentially corrects for differences in T_1_ values on different scanners and sequences, making it appealing as a technique for multicenter research.

A number of clinical studies have validated ECV% against histology in aortic stenosis and have demonstrated the association between ECV% and other markers of LV decompensation, including ECG changes of hypertrophy and strain and elevation in biomarkers such as troponin and N-terminal pro-brain natriuretic peptide 26, 39, 40, 41 (Table 2). ECV% also demonstrates excellent scan-rescan reproducibility (59), while guidelines to standardize post-processing have been developed and recommend that areas of noninfarct LGE are included and areas of infarct LGE excluded from regions of interest in ECV% calculation (69). However, data assessing the prognostic value of ECV% in aortic stenosis are limited, and overlap between disease groups is again observed. In addition, the effect of AVR on ECV% may be somewhat counterintuitive as values can increase after surgery—a weakness of assessing the extracellular component of the myocardium as a fraction of the ventricular mass when both the intracellular and extracellular compartments are undergoing reverse remodeling (25).

#### Indexed extracellular volume

**Figure 3:**
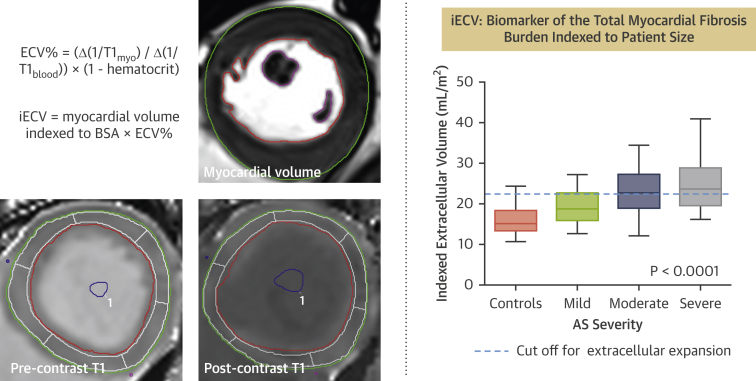
iECV calculation The cardiac magnetic resonance short-axis images provide examples of the pre-contrast and post-contrast contours required to calculate iECV. Systolic and diastolic contours are drawn using the short-axis stack to calculate myocardial volume, which is necessary to derive iECV. Color look-up tables have not been applied to the T_1_ images. iECV provides a surrogate of the total myocardial fibrosis burden according to the formula demonstrated in the figure. iECV demonstrates good correlation with histological fibrosis burden and severity of aortic stenosis. Graph adapted from Chin et al. (41), Creative Commons Attribution License: https://creativecommons.org/licenses/by/4.0/. BSA = body surface area; other abbreviations as in Figure 2.

Whereas ECV% provides a percentage estimate, the indexed extracellular volume (iECV) quantifies the total left ventricular extracellular myocardial volume indexed to body surface area by multiplying ECV% by the indexed left ventricular myocardial volume: iECV = ECV% × indexed left ventricular myocardial volume (Figure 3). Furthermore, cellular volume can be calculated: (1 − ECV%) × left ventricular volume). This can also be indexed to body surface area. In combination with LV mass, ECV% and iECV can together provide an understanding of ventricular remodeling and reverse remodeling with respect to both the cellular and extracellular myocardial compartments. Two studies have utilized iECV or matrix volume as a novel assessment of myocardial fibrosis burden 25, 41, with iECV demonstrating a close association with histological fibrosis assessments. Importantly, iECV appears to provide greater discrimination between disease states than other T_1_ mapping parameters. Chin et al*.* (41) demonstrated that a threshold of 22.5 ml/m^2^ (derived from 37 age- and sex-matched healthy volunteers and defined as 2 SDs above the mean) could be used to differentiate healthy myocardium from diseased myocardium infiltrated by diffuse fibrosis, and in doing so, identify patients with early evidence of left ventricular decompensation and adverse long-term outcome (41).

**Figure 4:**
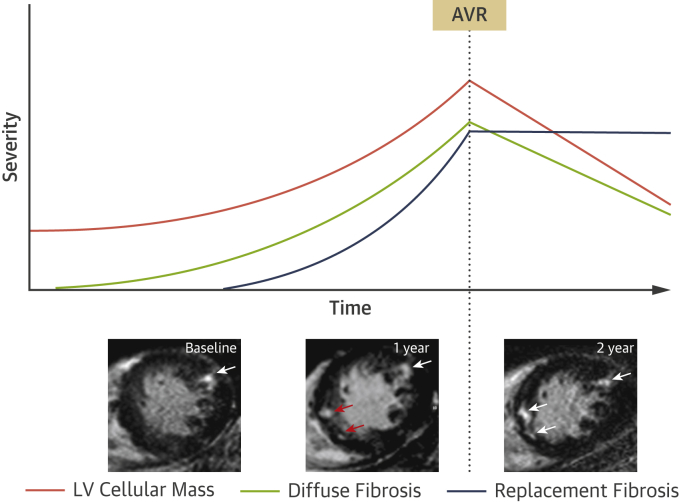
Schematic for the Development of Myocardial Fibrosis in Aortic Stenosis and Response to AVR As aortic stenosis progresses, left ventricular (LV) mass gradually increases, followed by the development of diffuse fibrosis. Replacement fibrosis occurs later but accelerates rapidly once established. Following relief of pressure-loading conditions after aortic valve replacement (AVR), LV cellular mass and extracellular matrix both regress at different rates. The burden of replacement fibrosis, however, persists. The **insets** show short-axis cardiac magnetic resonance late gadolinium enhancement imaging slices of a patient with aortic stenosis. At baseline, there is focal late gadolinium enhancement representing discrete focal replacement fibrosis **(white arrow)**. After 1 year, the burden of this replacement fibrosis has increased with the development of several new discrete deposits **(red arrows).** The patient subsequently underwent AVR. One year later, despite regression of LV mass, there is no regression of replacement fibrosis **(white arrows)**.

iECV and ECV% have recently been used in combination to study changes in the composition of the intracellular and extracellular compartments before and after AVR. This has provided important insights into left ventricular remodeling and reverse remodeling after relief of loading conditions. Changes in iECV are not accounted for by changes in total left ventricular mass alone. Prior to AVR, iECV (representing total extracellular matrix, or fibrosis, burden) and left ventricular mass appear to increase in a broadly balanced manner so that ECV% remains largely unchanged. Following AVR, left ventricular mass decreases. Cellular and extracellular mass regress, but cellular mass regresses more rapidly, thereby resulting in an apparently paradoxical increase in ECV% as the ratio of matrix to total mass is increased 25, 42. iECV, however, decreases as it represents the extracellular matrix as a total volume, rather than a percentage. The reduction in iECV is therefore in keeping with the potential for reversal of diffuse fibrosis. This effect has been confirmed independently by 2 different groups in separate cohorts and stands in contrast to the irreversible nature of replacement fibrosis as assessed by LGE (Figure 4). iECV requires further exploration and validation but is a promising method to track myocardial fibrosis.

In summary, T_1_ mapping is an exciting and emerging research field in aortic stenosis research that provides the only method of identifying reversible diffuse myocardial fibrosis. It holds particular potential as a method to track myocardial health over time, with important clinical implications. Standardization of sequences and protocols have resulted in reproducible and powerful prognostic T_1_ mapping data in a variety of myocardial disease states 41, 53, 54, 58. However, T_1_ mapping in aortic stenosis is in a relatively early stage of development. Further work is required to establish validated thresholds to aid decision making, paving the way for future multicenter prognostic studies that are ultimately required. Of the T_1_ mapping parameters currently in use, we believe that ECV% and iECV currently provide the most complete understanding of cellular and extracellular remodeling in aortic stenosis, although native T_1_ provides important advantages, particularly with regard to ease of calculation and the avoidance of contrast administration.

### Other imaging modalities

Alternative imaging techniques to assess myocardial fibrosis in aortic stenosis are limited. Research into computed tomography (CT) assessments of myocardial fibrosis remains exploratory, with only limited data available that has largely focused on measuring ECV on CT scans performed after the administration of iodinated contrast agents (Table 3). These techniques are worthy of further investigation given the widespread use of CT imaging in patients being considered for TAVR and the emerging utility of CT calcium scoring as a marker of stenosis severity. Strain imaging on echocardiography or CMR can be a valuable noninvasive tool to evaluate and quantify myocardial deformation before any identifiable changes in ejection fraction; however, despite an association with imaging markers of myocardial fibrosis 27, 29 and potential prognostic utility 70, 71, this approach is unable to measure myocardial fibrosis directly.

**Table 3:** CT to Detect Myocardial Fibrosis

Study (Ref. #)	Year	n	Population	CT	Biopsy	CMR	Findings
Bandula et al. (85)	2013	23	Severe AS undergoing SAVR	Iohexol equilibrium bolus and infusion protocol	23	shMOLLI	ECV_CT_ correlated with ECV_CMR_ (r = 0.73; p < 0.001) and histological fibrosis (r = 0.71; p < 0.001).
Hong et al. (86)	2016	20	Rabbits 4 healthy 16 DCM	Dual-energy CT Iopamidol bolus	20	3-T MOLLI	ECV_CT_ correlated with ECV_CMR_ (r = 0.89; p < 0.001) and histological fibrosis (r = 0.925; p < 0.001).
Treibel et al. (87)	2017	73	Validation cohort: 28 severe AS 27 amyloid 18 severe AS underdoing SAVR	64-detector Iohexol bolus	18	—	Good correlation between synthetic and conventional ECV_CT_ (r^2^ = 0.96; p < 0.001). Good correlation between synthetic and conventional ECV_CT_ and histology (both r^2^ = 0.50; p < 0.001). ECV_CT_ was higher in amyloidosis.
Nacif et al. (88)	2012	24	11 healthy 13 HF	320-detector Iopamidol bolus	—	3-T 3(3)5 MOLLI	Correlation between CMR and CT (r = 0.82; p < 0.001). ECV lower in healthy patients for both CMR and CT (p = 0.03).
Nacif et al. (89)	2013	24	9 healthy 10 HFrEF 5 HFpEF	320-detector Iopamidol bolus	—	—	Mean 3D ECV significantly higher in HFrEF than other groups (p = 0.02).
Treibel et al. (90)	2015	47	27 severe AS 26 amyloid	64-detector Iodixanol dynamic equilibrium bolus protocol	—	1.5-T shMOLLI	ECV_CT_ at 5 min and 15 min correlated with ECV_CMR_ (r^2^ = 0.85; r^2^ = 0.74; p < 0.001). ECV_CT_ was higher in amyloidosis and correlated with markers of severity.
Lee et al. (91)	2016	30	7 healthy 6 HCM 9 DCM 4 amyloid 4 sarcoid	Dual-energy CT Iopamidol bolus	—	3-T 3(3)5 MOLLI	Good agreement between ECV_CT_ and ECV_CMR_ on per-subject (Bland-Altman bias 0.06%; 95% CI: 1.19–1.79) and per-segment level.

CT = computed tomography; DCM = dilated cardiomyopathy; HF = heart failure; HFpEF = heart failure with preserved ejection fraction; HFrEF = heart failure with reduced ejection fraction; other abbreviations as in Table 2.

## Future Directions

Myocardial fibrosis is well established as a hallmark pathological feature of left ventricular decompensation in patients with aortic stenosis; yet, it is not routinely assessed in clinical practice. In part, this has reflected the limitations of myocardial biopsy, many of which have now been overcome with advanced noninvasive imaging. The next step is to assess whether these imaging techniques will prove of clinical value in monitoring myocardial health, identifying left ventricular decompensation, and optimizing the timing of AVR.

**Figure 5:**
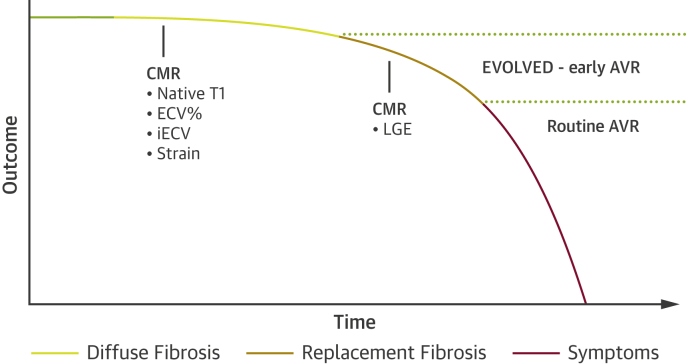
Proposed Integration of Myocardial Fibrosis Into the Classical Description of the Natural History of Aortic Stenosis Adaption of the outcome curve originally proposed by Braunwald in 1968 (76). Prior to the onset of symptoms, there is a long latent period in aortic stenosis where subclinical myocardial changes take place, including the development of reversible diffuse fibrosis followed by irreversible replacement fibrosis. These changes may be assessed with the imaging modalities denoted in the figure. Exploratory data suggest that diffuse fibrosis is associated with an adverse long-term outcome in aortic stenosis. The prognostic data related to the noninfarct pattern of late gadolinium enhancement (LGE) as a marker of replacement fibrosis is comparatively robust, establishing LGE as a powerful independent predictor of long-term clinical outcomes. According to current guidelines and routine clinical practice, AVR is performed after the onset of symptoms. Future and ongoing trials, including the EVOLVED trial, are required to determine whether targeted early intervention utilizing cardiac magnetic resonance (CMR) to detect fibrosis will lead to improved clinical outcomes. Abbreviations as in Figures 2 and 4.

LGE is the best validated of these approaches, is relatively simple to perform and analyze, and is supported by powerful prognostic data. Whether noninfarct LGE can be used to optimize the timing of valve intervention is currently being tested in the EVOLVED (Early Valve Replacement Guided by Biomarkers of LV Decompensation in Asymptomatic Patients With Severe AS) trial (NCT03094143) (47) (Figure 5). This multicenter randomized controlled trial will recruit asymptomatic patients with severe aortic stenosis for CMR imaging. Those patients with noninfarct LGE will then be randomized 1:1 to early valve intervention (SAVR or TAVR) versus the conventional approach of watchful waiting until symptom development or clinical heart failure. To mitigate the costs of CMR, patients will initially be screened with high-sensitivity troponin and an electrocardiogram, both of which are predictors of noninfarct LGE (72); only those patients with an abnormal electrocardiogram or a troponin ≥6 ng/l will proceed to CMR. The primary endpoint is a composite of all-cause mortality and unplanned aortic stenosis–related hospital admissions. This is the first randomized trial to offer targeted early intervention in patients with myocardial fibrosis and left ventricular decompensation, and the results will be of great interest. Similar randomized controlled trials will ultimately be required to establish the clinical utility of other myocardial fibrosis assessments, given that aortic valve intervention is not without risk.

CMR assessments of diffuse fibrosis in aortic stenosis require further validation but offer the potential to identify the earlier stages of myocardial disease and track myocardial health with time. T_1_ mapping is the only available imaging technique that is able to offer an assessment of diffuse fibrosis, and as such, it is crucial that ongoing research is conducted to provide standardization of sequences and protocols across sites and vendors to delineate clear cutoffs for health and disease in aortic stenosis. As T_1_ mapping research expands, this approach may offer clear advantages over LGE. For example, future investigation of antifibrotic therapies will require biomarkers to monitor myocardial health and treatment effects; T_1_ mapping will be indispensable in this regard.

Further work to investigate the role of emerging CT techniques is also warranted, particularly as they may be more easily integrated into current clinical care pathways and workflows than CMR. There has also been early investigation of collagen- and elastin-specific CMR contrast agents, which may provide greater contrast to noise ratio compared with current GBCAs, but further advances in this field are awaited 73, 74. Finally, there is considerable interest in developing novel positron-emission tomography tracers to measure myocardial fibrosis activity, in contrast to the structural and functional assessments that have been developed to date. We await further studies to demonstrate this potential. As interest in this field progresses and new techniques emerge, it is of course important to be cognizant of publication bias, which remains an issue in the published medical data (75).

## Conclusions

Myocardial fibrosis plays a key role in the pathophysiology of aortic stenosis. Modern imaging techniques now allow assessment of both replacement and diffuse interstitial fibrosis as well as their functional consequences. These techniques hold promise in tracking myocardial health in patients with aortic stenosis, aiding risk stratification and potentially optimizing the timing of aortic valve intervention, with ongoing trials currently testing the clinical efficacy of these approaches.
